# Social Behaviors Increase in Children with Autism in the Presence of Animals Compared to Toys

**DOI:** 10.1371/journal.pone.0057010

**Published:** 2013-02-27

**Authors:** Marguerite E. O'Haire, Samantha J. McKenzie, Alan M. Beck, Virginia Slaughter

**Affiliations:** 1 School of Psychology, The University of Queensland, Brisbane, Queensland, Australia; 2 School of Population Health, The University of Queensland, Herston, Queensland, Australia; 3 College of Veterinary Medicine, Center for the Human-Animal Bond, Purdue University, West Lafayette, Indiana, United States of America; Lancaster University, United Kingdom

## Abstract

**Background:**

Previous research has demonstrated the capacity of animal presence to stimulate social interaction among humans. The purpose of this study was to examine the interactions of children with autism spectrum disorder (ASD) with an adult and their typically-developing peers in the presence of animals (two guinea pigs) compared to toys.

**Methods:**

Ninety-nine children from 15 classrooms in 4 schools met the inclusion criteria and participated in groups of three (1 child with ASD and 2 typically-developing peers). Each group was video-recorded during three 10-minute, free-play sessions with toys and three 10-minute, free-play sessions with two guinea pigs. Two blinded observers coded the behavior of children with ASD and their peers. To account for the nested study design, data were analyzed using hierarchical generalized linear modeling.

**Results:**

Participants with ASD demonstrated more social approach behaviors (including talking, looking at faces, and making tactile contact) and received more social approaches from their peers in the presence of animals compared to toys. They also displayed more prosocial behaviors and positive affect (i.e., smiling and laughing) as well as less self-focused behaviors and negative affect (i.e., frowning, crying, and whining) in the presence of animals compared to toys.

**Conclusions:**

These results suggest that the presence of an animal can significantly increase positive social behaviors among children with ASD.

## Introduction

Autism spectrum disorder (ASD) is a prevalent and debilitating disorder estimated to affect up to 1 in 91 children in the US, with rates growing worldwide [1], [2]. The core feature of the disorder is impairment in social interaction and communication [3]. In the school environment, these social deficits can be particularly devastating. Children with ASD in mainstream or “inclusion” classrooms with their typically-developing (TD) peers often struggle to engage with their classmates and experience resultant social isolation, rejection, bullying, and stress [4]–[6]. These experiences can lead to inferior academic performance and problem behaviors [7]. In addition to low social engagement with peers in the inclusion classroom, children with ASD also engage in much less social interaction with teachers than their TD peers [8]. Less social engagement and more problem behaviors can lead to poorer teacher-student relationships among children with ASD [9] and contribute to higher rates of teacher burnout [10], [11]. Developing an innovative and effective strategy for children with ASD to improve social interaction with peers and adults has therefore become an important research priority [12]. One such strategy may be the incorporation of human-animal interaction (HAI) into the classroom environment [13].

Previous HAI studies have demonstrated the capacity of animals to encourage social interaction among humans. For example, when walking with a dog, people are more likely to receive positive social approaches from strangers than when walking alone [14]. A similar effect has been documented for individuals sitting with a rabbit or turtle on a park bench [15]. The “social lubricant” effect of animals can be particularly important for individuals with disabilities, for whom the presence of an animal can provide a normalizing effect and a conversation starter [16]. Interacting with animals may also offer a context for enhanced socio-emotional development [17]. For example, the introduction of an animal into the home of a child with ASD has been related to increased empathy and prosocial behavior [18]. The presence of a service dog in the home has also been related to increased mood and sense of well-being among children with ASD and their families [19]. The documented benefits of HAI have led to the practice of incorporating animals into therapeutic endeavors, known as Animal-Assisted Intervention [20]. Its use for individuals with ASD is the subject of growing scientific inquiry [21].

A recent systematic literature review identified 14 peer-reviewed studies that evaluated Animal-Assisted Intervention for individuals with ASD [22]. Of these, five studies used smaller animals (not horses) and evaluated child behavior in the presence of an animal compared to no animal. The most common finding among these studies was increased social interaction in the presence of the animal compared to sessions without an animal. Greater social interaction was defined by increased verbal social approach behaviors, such as talking to the therapist [23], [24] or talking about the animal [25], increased visual social approach behaviors, such as looking at the therapist [26], and increased overall social behaviors, including a composite of verbal, visual, and physical (tactile) social approach behaviors towards ASD peers and teachers [27]. Additional findings included increased positive emotional displays in the presence of the animal, such as more smiling [26] and laughing [25], as well as decreased problem behaviors, including aggression, grabbing [26], and social isolation, which was defined as play or self-stimulatory activities directed to the self [24].

Despite positive findings, these studies were subject to a number of limitations. For example, in the condition without an animal, only one study introduced an alternative focus of attention (i.e., a ball or a stuffed dog [25]), while the others provided the same environment with no animal. Findings may therefore have been attributed to the addition of an attentional focus, rather than the animal specifically [28]. In addition, only one study examined interactions with peers without the intervention of a trained therapist [27], while the other four examined social behaviors directed to an adult therapist in the context of targeted therapeutic activities. The combination of an animal with therapeutic activities rather than the effects of the animal alone may account for the results. In addition, the study including peer targets examined interactions with other ASD peers and teachers in a special education classroom, but did not distinguish between interactions towards peers versus the adult and did not evaluate interactions with TD peers. Finally, none of the studies used blind observers of behavior, which may have led to biased outcomes. Instead, raters included the author, research staff or students, as well as unspecified personnel involved in the research.

In the present study, we build upon the current research base with the first blinded ratings HAI among children with ASD. We also present a comparison of interactions in the presence of an animal to interactions in the presence of an alternative focus of attention, namely a motivating collection of toys. Toys were selected as the attention control because they have been documented as an effective means of promoting interaction among children with ASD [29]. Further, the current study evaluates interactions with TD peers in a naturalistic environment without therapeutic intervention in order to gauge the influence of animals independent of targeted intervention. We also expanded upon previous coding systems by designing a comprehensive behavioral coding system to evaluate social approach behaviors (verbal, visual, and physical), prosocial behaviors, problem behaviors, and emotional displays. Targets of social approach behaviors are incorporated into the system in order to determine which humans the child with ASD interacts with (adult or TD peer) as well as whether there are differences between interactions with toys versus animals. The current study is also novel in that our coding system concurrently evaluates the social approaches of TD peers towards the child with ASD.

Based on previous HAI findings, our primary hypothesis was that children with ASD would demonstrate increased social approach behaviors, prosocial behaviors, and positive emotional displays, as well as decreased problem and self-focused behaviors in sessions with an animal, compared to sessions with toys. We also hypothesized that TD peers would demonstrate increased social approach behaviors towards children with ASD in the presence of animals when compared to the presence of toys.

## Methods

### Ethics Statement

All human-related and informed consent protocols were approved by The University of Queensland's Human Ethics Committee and all animal-related protocols were approved by The University of Queensland's Animal Ethics Committee. Approval to approach school principals was granted by the Queensland Department of Education, Training, and Employment for state schools and Brisbane Catholic Education for private schools. Upon written consent from the principal, teachers and parents (including next of kin or guardians) were approached for written consent on behalf of child participants, who also gave verbal assent.

### Participants

#### Recruitment and Eligibility

Participants were recruited from primary schools in the greater Brisbane area. Inclusion criteria for target participants with ASD included: (a) age between 5 to 13 years; (b) enrolment in a grade K-7 inclusion classroom, (c) a parent- and teacher-reported diagnosis of ASD, including Autism Spectrum Disorder, Autistic Disorder, Asperger's Disorder, or Pervasive Developmental Disorder Not Otherwise Specified (PDD-NOS), and (d) no prior parent-reported history of animal abuse. Following data collection, inclusion criteria for data analysis was based on two ASD screening instruments, and included: (a) a score ≥11 on the Social Communication Questionnaire (SCQ) to indicate the presence of ASD [30] and (b) a percentile rank ≤25 on the Social Skills Rating System (SSRS) Social Skills domain parent- or teacher-version to indicate low social skills characteristic of ASD [31]. Alternatively, in the absence of SCQ data or an SCQ score <11, the inclusion criterion was set to a more stringent percentile rank of ≤5 on SSRS Social Skills. Inclusion criteria for typically-developing peers included: (a) age between 5 to 13 years; (b) enrolment in a classroom with a target participant with ASD, (c) no previous diagnosis of ASD, and (d) no prior parent-reported history of animal abuse.

#### Sample Characteristics

Thirty-eight groups of three children (114 children total) participated in the study. Each group consisted of one target participant with ASD and two TD peers. Following data collection, five groups were excluded from data analysis for the following reasons: (a) the child with ASD changed schools after the first session, (b) one of the TD peers decided that they did not want to be video recorded after the second session, and (c) three participants with ASD did not meet the screening criteria for ASD on the SCQ and SSRS. The final sample included 33 groups with 99 children total. Study participants were spread across 15 inclusion classrooms in four different mainstream schools throughout the greater Brisbane area in Australia.

Target participants with ASD included 33 children (24 male; 9 female) aged 5.2 to 12.1 years (*M* = 9.4; *SD* = 2.3) in kindergarten (preparatory year) through seventh grade. All had a previous diagnosis of ASD, including Autism Spectrum Disorder (*n* = 7), Asperger's Disorder (*n* = 14), Pervasive Developmental Disorder Not Otherwise Specified (*n* = 5), and Autistic Disorder (*n* = 7). Diagnoses of ASD were made by pediatricians (*n* = 30), clinical psychiatrists (*n* = 2), and clinical psychologists (*n* = 1). On the SCQ, 18 participants qualified for ASD and 9 qualified for autism. The remaining participants (three scoring <11 and three missing SCQ data) all scored a percentile rank ≤5 on SSRS Social Skills.

The sample of TD peers included 66 children (28 male; 38 female) aged 5.1 to 12.7 years (*M* = 9.0; *SD* = 2.3). None had a prior diagnosis of ASD and none met the criteria for ASD or autism on the SCQ (all scores ≤10). Mean participant demographic data and outcomes of ASD screening measures are reported in Table 1.

**Table 1:** Demographic information and ASD screening measures.

Variable	Group				
	ASD		TD		ASD vs. TD
	*n*	%	*n*	%	*p*
Demographics					
Sex (male)	33	72.7%	66	42.4%	.004
Pet owners	33	81.8%	59	72.9%	.214
		***M (SD)***		***M (SD)***	
Age (years)	33	9.4 (2.3)	66	9.0 (2.3)	.465
ASD Screening Measures					
SCQ Lifetime	30	18.9 (6.6)	60	3.7 (2.7)	<.001
SSRS Social Skills					
Teacher-version	33	24.4 (24.9)	64	72.9 (28.7)	<.001
Parent-version	32	6.9 (12.2)	59	53.3 (28.0)	<.001
SSRS Problem Behaviors					
Teacher-version	33	75.6 (23.0)	64	38.1 (27.6)	<.001
Parent-version	32	86.1 (19.8)	59	45.5 (26.9)	<.001
SSRS Academic Competence					
Teacher-version only	33	26.0 (27.8)	64	50.9 (25.3)	<.001

ASD  =  autism spectrum disorder, TD  =  typically-developing, SCQ  =  Social Communication Questionnaire, SSRS  =  Social Skills Rating System.

### Measures

Two standardized instruments were administered for ASD screening purposes.

#### Social Communication Questionnaire (SCQ)

The SCQ, formerly known as the Autism Screening Questionnaire, is a 40-item parent-report screening questionnaire for ASD [32]. It the most researched and well validated parent-report screening tool for ASD [30]. The SCQ was designed based on the Autism Diagnostic Interview-Revised (ADI-R) [33]. Items on the SCQ correspond to criteria used to diagnose the core features of ASD through the Diagnostic and Statistical Manual 4^th^ Edition (DSM-IV), including communication, reciprocal social interactions, and repetitive behaviors and interests [3]. The instrument has excellent agreement with the ADI-R [34] as well as the DSM-IV criteria for diagnosis of autism [35]. It demonstrates good reliability and validity, and shows strong discrimination between ASD and non-ASD cases (sensitivity .88–.92, specificity, .62–.72), irrespective of child IQ or parental education [36], [37].

The lifetime version (for children over the age of 5 years) was used in the current study. Each item on the SCQ is rated as “yes” or “no” and assigned a 0–1 point rating (0 =  absence of abnormal behavior, 1 =  presence of abnormal behavior). Items address both current and past behavior. The possible range of scores for nonverbal children is 0–33 and for verbal children is 0–39. The cutoff scores used for ASD screening purposes are ≥11 for ASD and ≥22 for autism [30]. Complete SCQ data were obtained from 91% (*n* = 90) of parents.

#### Social Skills Rating System (SSRS)

The SSRS is a 57-item (elementary level teacher version) and 55-item (elementary level parent version) questionnaire designed to assess overall social skills in children with or without a clinical diagnosis [38]. It is commonly used to assess social functioning and demonstrates adequate internal consistency and test-retest reliability [38]. It is divided into two broad behavioral domains, including: (1) *Social Skills* (subscales include Cooperation, Assertion, Responsibility, and Self-Control) and (2) *Problem Behaviors* (subscales include Externalizing, Internalizing and Hyperactivity). The teacher version does not include the Responsibility subscale, but does include an additional *Academic Competence* subscale.

SSRS behavioral items are rated on a 0–2 scale of how often the child demonstrates a given behavior (0 =  never, 1 =  sometimes, 2 =  very often). SSRS Academic Competence items are rated on a 1–5 scale of how favorable their performance is compared to other students in the same classroom (1 =  lowest 10%, 2 =  next lowest 20%, 3 =  middle 40%, 4 =  next highest 20%, 5 =  highest 10%). The SSRS provides standard scores (*M* = 100; *SD* = 15) and percentile ranks for each domain based on age- and gender-specific norms [38]. Higher scores on SSRS Social Skills and SSRS Academic Competence represent better social functioning and academic functioning respectively, while lower scores on SSRS Problem Behaviors indicate better behavioral functioning. Given diagnostic deficits in social skills associated with ASD, the SSRS has been demonstrated as an effective tool to differentiate individuals with ASD from TD individuals [31], [39]. Cutoff scores for the current study were set to include only participants scoring in the lower quartile (percentile rank ≤25) of the SSRS Social Skills domain on either the parent- or teacher-version. Complete SSRS data were obtained from 98% (*n* = 97) of teachers and 92% (*n* = 91) of parents.

### Procedures

The current experiment took place as part of a larger study to examine the impact of Animal-Assisted Activities (AAA) on children with ASD in inclusion classrooms. The overall study comprised of an eight-week waitlist period followed by an eight-week AAA program where guinea pigs lived in the school classroom. The study start was staggered across schools over the course of one school year. The AAA program consisted of two guinea pigs living in each participating classroom, combined with twice-weekly, take-out sessions with the animals for each participant group (including one child with ASD and two TD peers). Take-out sessions took place outside of the regular classroom each week and were provided for the purpose of ensuring at least 40 minutes of contact time with the animals per week. Each 20-minute session followed an open-ended, child-directed structure. The AAA program was not a therapeutic intervention and had no targeted treatment goals. Instead, it was intended to evaluate the influence of animals in the classroom without the clinical components of Animal-Assisted Therapy. During the program, participants engaged in both toy sessions and animal sessions, as detailed below. During the final toy session, participants were asked which activity they preferred: reading, toys, or guinea pigs.

#### Program Facilitator

All sessions took place under the supervision of the program facilitator, one of the researchers (MEO). Prior to the first session, the facilitator met with each participant individually to familiarize participants with herself and the experiment. The initial meeting was also intended to reduce potential novelty effects of a new person during the first session. The role of the facilitator was to introduce the session items (toys or animals) and ensure both child and animal safety and welfare. The facilitator was also available to provide information regarding toys (e.g., how to play with a game) and animals (e.g., how to hold an animal) as needed. During sessions, the facilitator sat on the floor alongside the children to be easily accessible.

#### Toy Sessions

Toy sessions consisted of a set of standardized toys presented to children for unstructured interaction time. They took place at three time points throughout the larger study, including (1) upon study entry during the week prior to the eight-week waitlist period, (2) during the week following the eight-week waitlist period, and (3) during the week following the eight-week AAA program. Toy sessions were only conducted on days when all three participants from a given group were present at school. If one or more were absent, the session was rescheduled for the next available day. A variety of toys were selected to suit a range of ages and both male and female participants. The sample of toys included markers, colored pencils, one blank drawing book, blank paper, one coloring book, two spinning tops with rip-cord launchers for use in a plastic battle arena (Beyblade™), two fashion dolls (Moxie Girlz™), two fashion design art kits for the dolls (Art-titude™) including erasable markers to draw on a set of clothing and accessories, a restaurant set of 50+ plastic pieces (e.g., food, cutlery, menu, serving tray, apron, money), a set of 80+ multicolored toy building bricks, two paddle-ball games (i.e., paddle and ball attached by rubber string), multicolored modeling material (Play-Doh™) with modeling tools (e.g., shape cutters, rolling pin), bubble liquid and one blower, two toy cars, and one slinky.

#### Animal Sessions

Animal sessions consisted of two guinea pigs and animal-related materials presented to children for unstructured interaction time. Three animal sessions were selected for video coding from the set of sessions in which all three participants from a given group were present, including (1) the first session, (2) the last session, and (3) a randomly selected session from the remaining sessions. The two guinea pigs were the current classroom pets, which lived in the classroom for the duration of the eight-week AAA program. The total sample of animals included 30 guinea pigs ranging in age from four to eight weeks at the start of the program. Guinea pigs were housed in same sex pairs (two per classroom) for the duration of the study to prevent breeding and provide social enrichment for the animals. Animal-related materials in each session included guinea pig food (e.g., fruit, vegetables, pellets), towels, weighing scale, measuring tape, camera, markers, colored pencils, blank notebook, health checklist, recycled materials for building houses and mazes (e.g., cardboard or tissue boxes), scissors, glue, string, baby brushes for grooming, bathing supplies (e.g., small animal shampoo), and cage cleaning supplies (e.g., cleaning solution, paper towels, fresh bedding).

#### Video Recording

All toy and animal sessions were video recorded for later coding. The video camera was positioned approximately 15 feet in front of the session materials on a tripod, with the focal length adjusted to closely frame all participants. It was monitored and adjusted by a research assistant, in order to ensure that participants were in view at all times.

### Behavioral Coding

#### Sampling

Six sessions (three with toys and three with animals) were assessed for each participant group (198 sessions total). The first 10 minutes of each selected session were isolated for coding. In toy sessions, the 10 minutes started upon presentation of the toys, at the moment in which the sheet covering the toys was removed. In animal sessions, the 10 minutes started upon presentation of the animals, at the moment in which the first guinea pig was removed from the fenced area in front of participants.

Within each 10-minute segment, three minutes were selected for coding (594 minutes total) using a timed interval sampling procedure [40]. We replicated the protocol enlisted in previous HAI research [25] by coding one minute from the first third, one minute from the second third, and one minute from the last third of each session. Minutes within each third were randomly selected.

#### Coders

Two independent, blind observers were trained in the coding procedure. Observers were blinded to the study aims, design, hypotheses, analyses, and outcomes. The primary coder was a psychology graduate student with extensive experience in behavioral coding of children with ASD. The secondary coder was a psychology undergraduate student. The primary coder rated 100% of selected segments and the secondary coder rated 40% of selected segments for reliability. Inter-rater reliability was evaluated using Cohen's Kappa [41], which indicated excellent overall agreement among raters (*k* = .79, *p*<.001) [42]. Reliability was also calculated for specific behavioral categories, including social approach behaviors (*k* = .73, *p*<.001), received social approaches from peers (*k* = .64 *p*<.001), interaction with toys/animals (*k* = .91, *p*<.001), other behaviors (*k* = .90, *p*<.001), emotional displays (*k* = .74, *p*<.001), and verbal valence (*k* = .62, *p*<.001).

#### Behavior Coding System

The Observation of Human Animal Interaction for Research (OHAIRE) is a timed interval coding system that was designed for the purposes of this study. It was developed based on previously published behavioral codes of children with ASD in the classroom setting [29], [43], [44] and children with ASD during interaction with animals [23]–[27]. It includes codes for social behaviors, including verbal, visual, and physical approaches. It also includes codes for prosocial behaviors, problem behaviors, and emotional displays. Social behaviors are primarily coded for participants with ASD, but targeted social approaches from TD peers are also coded. The definitions of each behavioral code are detailed in Appendix S1.

The OHAIRE coding system involves coders rating the presence or absence of each behavior during 10-second intervals of a selected minute. Each interval is watched twice in succession. On the first viewing, behaviors of the target participant with ASD are coded. On the second viewing, behaviors of TD peers directed at the participant with ASD are coded. The resultant score for each behavioral code is the number of 10-second intervals within a minute, in which the behavior occurred. In order to reduce data entry error associated with paper-based collection instruments, behavioral codes were recorded on an iPad through an internet-based OHAIRE coding program designed on Qualtrics Online Survey Software.

### Data Analysis

Prior to examining the primary hypotheses, we checked for differences between ASD and TD participants on the two ASD screening measures (SCQ raw scores and SSRS percentile ranks) in order to provide additional validation of parent-reported ASD diagnoses. We also checked for differences on potentially confounding demographic variables, including age, gender, and pet ownership status. Independent samples *t* tests were conducted for continuous variables (i.e., age, SCQ Lifetime, SSRS Social Skills, SSRS Problem Behaviors, SSRS Academic Competence) and Chi-square tests were used for categorical variables (i.e., gender, pet ownership status) with a significance level cut-off of α = 0.05.

In order to account for the nested study design (i.e., multiple assessments nested within individuals nested within classrooms nested within schools) and count data as the outcome variable (i.e., number of intervals per minute in which a behavior occurred), we used hierarchical generalized linear modeling (HGLM) for data analysis of our primary hypotheses. HGLM, or generalized linear mixed modeling, offers an effective procedure for nested, longitudinal, non-linear, and non-normal data [45]. For most models, we conducted the standard HGLM for count data by specifying a Poisson distribution sampling model with a log-link function [46]. For outcome variables with overdispersion, we specified a negative binomial sampling model with a log-link function [47]. We used the generalized linear mixed model procedure available within the Statistical Package for the Social Sciences (SPSS) Version 20.0 [48].

We conducted a series of four-level HGLMs, where the levels reflected repeated measurements (Level 1), individual effects (Level 2), classroom effects (Level 3), and school effects (Level 4). Random effects in the model were identified as the repeated measures effect of time (to account for correlations between repeated observations of the same participant) as well as intercepts at the individual-level (to account for variance across individuals), classroom-level (to account for correlation between individuals in the same classroom), and school-level (to account for correlation between classrooms within the same school).

We addressed our primary hypothesis by including the fixed effect of session type (toy or animal). In order to control for potential covariates and their interactions with session type, we included the additional fixed factors of grade, pet ownerships, SCQ score, and the interaction between each of these factors and session type. To account for three missing data points on the SCQ due to parents not completing the instrument, we used maximum likelihood estimation using the expectation-maximization (EM) algorithm [49] as the recommended method for handling missing data [50]. Following EM estimation, continuous variables (i.e., grade and SCQ score) were grand-mean centered prior to HGLM analyses. All significance tests were two-tailed with a significance level of α<0.05. Reported effect sizes are Cohen's *d* [51].

## Results

### Preliminary Analyses: ASD Screening

Participant scores on the two ASD screening instruments confirmed differences between participants with ASD and their TD peers (Table 1). Participants with ASD scored significantly higher on the SCQ, *t*(88) = −15.49, *p*<.001, indicating a greater presence of abnormal behaviors on the autistic spectrum. On SSRS Social Skills, participants with ASD scored lower than their TD peers on both the teacher-version, *t*(95) = 8.24, *p*<.001, and the parent-version, *t*(89) = 8.91, *p*<.001. Thus, they were reported to exhibit fewer socially skilled behaviors than their TD peers. Participants with ASD scored higher on SSRS Problem Behaviors on both the teacher-version, *t*(95) =  −6.70, *p*<.001, and the parent-version, *t*(89) = −7.52, *p*<.001, indicating that they were reported to exhibit poorer behavioral functioning than their TD peers. Participants with ASD also scored lower on SSRS Academic Competence than their TD peers, *t*(95) = 4.44, *p*<.001, indicating lower academic performance compared to their TD peers. Taken together these findings are consistent with the parent-reported, independent diagnoses of ASD, in showing that the diagnosed children differed from their TD peers on many of the behavioral characteristics used to screen for ASD, including social communication, social skills, and behavioral functioning.

With respect to demographic characteristics, there was a higher proportion of males among the ASD participants than the randomly-selected TD participants, *X* ^2^(1, *N* = 99) = 8.10, *p* = .004. However, there were no significant differences between ASD and TD participants with respect to age, *t*(89) = −0.73, *p* = .465, or pet-ownership status *X* ^2^(1, *N* = 91) = 1.54, *p* = .214.

### HGLM Random Effects

The four-level HGLMs we conducted accounted for within-participant variance across repeated assessments (Level 1), between-participant variance across individuals (Level 2), between-classroom variance (Level 3), and between-school variance (Level 4). Results showed that the random effects of school (ICC's<.81, *p*'s>.243) and classroom (ICC's<.12, *p*'s>.258) were not significant in any models. Thus, there was no significant variability in outcomes across schools or classrooms. However, results showed that the random effects of between-participant variance (ICC range: .01–.68, *p* range: .001–.101) and within-participant variance (ICC range: .03–.93, *p* range: <.001–.300) were significant in most models. These findings indicate that the use of hierarchical models was appropriate in order to account for heterogeneity across individual participants and individual measurements within participants.

### HGLM Primary Outcomes: Toy vs. Animal

#### Social approach behaviors

**Figure 1:**
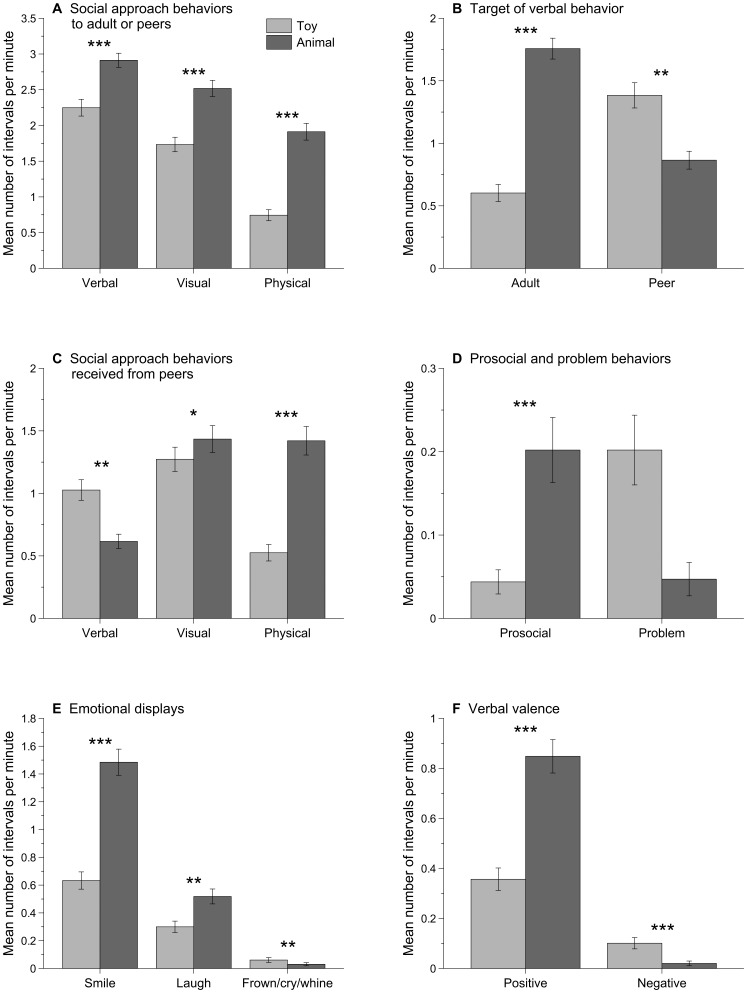
Observed behaviors during toy sessions (in light grey) and animal sessions (in dark grey). Values represent mean number of 10-second intervals per minute and error bars are standard error mean.

Participants with ASD displayed social approach behaviors during more 10-second intervals per minute in the presence of animals compared to toys (*p*<.001; Table 2). When broken down into three types of social approach behaviors, we found the same pattern for verbal (*p*<.001), visual (*p*<.001), and physical (*p*<.001) social approach behaviors (Figure 1A). Therefore, participants with ASD talked more, looked more at human faces, and made more tactile contact with people in the presence of animals compared to toys.

**Table 2:** Effect of toy versus animal presence on behavioral outcomes.

Variable	β (SE)	*t*	*d*
Social approach behaviors			
Overall	0.31 (0.05)	6.82***	1.23
Verbal	0.23 (0.06)	4.11***	0.56
Visual	0.33 (0.42)	5.24***	0.42
Physical	1.00 (0.68)	8.24***	0.68
To adult			
Overall	1.03 (0.08)	13.15***	1.16
Verbal	1.11 (0.11)	10.25***	0.88
Visual	0.87 (0.11)	8.09***	0.67
Physical	2.04 (0.35)	5.83***	0.50
To peers			
Overall	0.18 (0.06)	3.02**	0.25
Verbal	−0.40 (0.11)	−3.48**	0.34
Visual	0.20 (0.08)	2.48*	0.19
Physical	0.87 (0.15)	5.91***	0.52
Received approaches from peers			
Overall	0.26 (0.07)	3.83***	0.26
Verbal	−0.38 (0.13)	−2.90**	0.33
Visual	0.21 (0.08)	2.55*	0.09
Physical	1.01 (0.16)	6.48***	0.56
Interaction with toys/animals			
Overall	−0.10 (0.04)	−2.48*	0.37
Verbal	0.20 (0.12)	1.71	0.63
Visual	−0.13 (0.04)	−3.28**	0.43
Physical	−0.39 (0.04)	−9.16***	0.83
Verbal topic	0.07 (0.07)	0.96	0.09
Other behaviors			
Prosocial behaviors	0.66 (0.12)	5.47***	0.31
Self-focused behaviors	−2.75 (0.11)	−24.77***	4.93
Problem behaviors	−0.01 (0.12)	−0.12	0.27
Emotional displays			
Smile	0.88 (0.12)	7.49***	0.62
Laugh	0.57 (0.18)	3.08**	0.27
Frown/cry/whine	−0.50 (0.17)	−2.97**	0.12
Verbal valence			
Positive	0.86 (0.15)	5.95***	0.50
Negative	−0.74 (0.16)	−4.67***	0.27

β =  coefficient for animal sessions (reference: toy sessions), SE =  standard error, *d* =  Cohen's *d* effect size,

* *p*<.05,

** *p*<.01,

*** *p*<.001.

The target of social approach behaviors included either the adult or two TD peers. Overall, participants with ASD demonstrated social approach behaviors to adults (*p*<.001) and peers (*p* = .003) during more intervals per minute in the presence of animals compared to toys. For adult targets, when broken down into three types of social behavior, we found the same pattern for verbal (*p*<.001), visual (*p*<.001), and physical (*p*<.001) social approach behaviors. For peer targets, we found the same pattern for visual (*p* = .013) and physical (*p*<.001) social approach behaviors. However, we found a different pattern for verbal social approach behaviors (*p* = .003), in that participants with ASD talked to their peers during more intervals per minute in the presence of toys compared to animals (Figure 1B).

Therefore, participants with ASD displayed more overall social approach behaviors towards adult and peer targets in the presence of animals compared to toys. Specifically, they looked more at the faces of the adult and their peers and made more tactile contact with the adult and their peers in the presence of animals compared to toys. However, although they talked more to the adult in the presence of the animals, they talked more to their peers in the presence of the toys.

#### Received social approach behaviors from TD peers

TD peer participants displayed social approach behaviors towards the child with ASD during more intervals per minute in the presence of animals compared to toys (*p*<.001). When broken down into three types of behaviors, we found the same pattern for visual (*p* = .011) and physical (*p*<.001) social approach behaviors, but a different pattern for verbal behaviors (*p* = .004; Figure 1C). Therefore, participants with ASD received more overall social approaches from TD peers in the presence of animals. TD peers looked at their faces and made tactile contact more often in the presence of animals. However, they talked to children with ASD more often in the presence of the toys.

#### Interaction with toys versus animals

Participants with ASD engaged in overall interactions with the toys during more intervals per minute than they engaged in interactions with the animals (*p* = .014). Specifically, although there was a trend towards talking to the animals more often than the toys (*p* = .088), they looked at the toys significantly more often (*p* = .001) and touched the toys with their hands more often (*p*<.001) than the animals. However, there were only two animals compared to a variety of toys, so this effect may be a function of the availability of toys versus animals. When talking to other targets such as the adult or peers, there were no significant differences in how often they talked *about* the toys or the animals (*p* = .339). Participants with ASD did, however, demonstrate affection (e.g., hugging, cuddling, nuzzling, or comforting) to the animals during an average of 1.19 (*SE* = 0.11) intervals per minute. There were no instances of human-directed affection to the adult or peers in either condition. When asked whether they preferred reading, toys, or the guinea pigs, 81.8% of children with ASD indicated that they preferred the guinea pigs, followed by toys (12.1%), or both (6.1%). Therefore, participants with ASD interacted more with the toys than the animals, but most often preferred and displayed affection to the animals.

#### Prosocial, self-focused, and problem behaviors

Participants with ASD displayed prosocial behaviors (e.g., activities intended to benefit either their peers or the adult) during more intervals per minute in the presence of animals compared to toys (*p*<.001; Figure 1D). In contrast to prosocial behaviors, participants with ASD engaged in self-focused activities (e.g., play or self-stimulatory behaviors directed to the self) during more intervals per minute when with toys than with animals (*p*<.001). Thus, participants engaged in more other-focused activities during the animal sessions and more self-focused activities during the toy sessions. There were no significant differences in problem behaviors between the toy and animal conditions (*p* = .904).

#### Emotional displays

Participants with ASD smiled (*p*<.001) and laughed (*p* = .002) during more intervals per minute in the presence of animals compared to toys (Figure 1E). They also demonstrated fewer instances of negative affect (*p* = .003), including frowning, crying, and whining, in the presence of animals compared to toys. When speaking, the valence of verbal content was overtly positive (e.g., expressions of joy, liking, or happiness) during more intervals per minute in the presence of animals compared to toys (*p*<.001; Figure 1F). Additionally, the valence of verbal content was overtly negative (e.g., expressions of discontent, complaints, disliking, or sadness) during fewer intervals per minute in the presence of animals compared to toys (*p*<.001). Therefore, participants with ASD displayed more positive affect (and less negative affect) and talked more about positive things (and less about negative things) in the presence of animals compared to toys.

## Discussion

This study presented the first blinded observational ratings of children with ASD in the presence of animals compared to toys. Results supported our primary hypothesis that children with ASD would display more social behaviors in the presence of animals. In particular, children with ASD talked more to people, looked more at human faces, and made more tactile contact with humans in the presence of two guinea pigs compared to a selection of toys. They also received more social approaches from their TD peers in the presence of animals compared to toys. Further, participants with ASD showed more prosocial behaviors, displayed positive affect such as smiling and laughing more often, and displayed less negative affect in the presence of animals compared to toys. All outcomes were independent of differences across schools, classrooms, individuals, grade level, pet ownership, SCQ score, and repeated measurements over time. Taken together, the results suggest that the presence of an animal can facilitate increased positive social interaction for children with ASD.

The current study expands upon previous studies by demonstrating that the presence of an animal can stimulate social interaction above and beyond the presence of another social stimulus–toys. Additionally, while previous studies have reported increases in social behaviors during therapeutic sessions with animals (e.g., [23], [24]), the current study demonstrates that the presence of an animal alone, without concurrent therapeutic protocols, can increase social interaction. These findings support the rationale for including animals in therapy as a means of increasing engagement and interaction with therapists and practitioners [52]. In the current study, children with ASD displayed more social approach behaviors towards the adult in the presence of animals compared to toys. These social approaches included speaking to the adult, looking at the adult's face, and coming into physical contact with the adult. The ability of the animal to connect children with ASD to an adult in these ways may be conducive to building rapport and fostering interaction with a therapist, teacher, or other adult figure. An animal may therefore be a productive choice for play- or object-based interventions [53] in order to provide opportunities for social and communicative learning and engagement.

Children with ASD also displayed more social approach behaviors towards their TD peers in the presence of animals compared to toys. Specifically, they looked more at the faces of their peers and engaged in more physical contact with their peers. These findings are consistent with previous research demonstrating increases in the social approach behaviors of nine children with ASD in the presence of a guinea pig in one special education classroom [27]. The current study extends this research by replicating the findings with a larger sample size of 33 children with ASD in 15 regular education classrooms. It also includes the first evaluation of the social approaches of TD children towards the target child with ASD. In line with our hypothesis, we found that TD children also displayed increased overall social approach behaviors towards children with ASD in the presence of animals compared to toys. It appears that the animals facilitated increased social interaction on both the part of the child with ASD and their TD peers. The increased social contact of TD peers may be of particular value in inclusion classrooms, where children with ASD are often rejected and victimized by their TD peers [4]. Our results suggest that the addition of an animal to a small group setting may be more effective for increasing some forms of social interaction, such as looking and touching, than presenting toys to stimulate child interaction.

For children with ASD, increases in classically social behaviors are an important and often difficult to achieve phenomenon. In particular, children with ASD characteristically avoid visual contact with human faces (e.g., [54]). The ability of an animal's presence to increase this behavior may be related to the ability of animals to make people and scenes appear less threatening [55], [56]. It may also be a by-product of the stress-reducing effects of animal presence. Previous research has demonstrated reductions in a physiological indicator of stress (i.e., cortisol awakening response) in children with ASD following the introduction of a service dog into the home [57]. Other studies have shown that the presence of an animal can moderate stress responses by reducing cardiovascular reactivity (e.g., [58]). For children with ASD, the school classroom can be a stressful and overwhelming environment due to social challenges and peer victimization [59]. If an animal can reduce this stress or artificially change children's perception of the classroom and its occupants, then a child with ASD may feel more at ease and open to social approach behaviors.

Changes in children's perceptions of the situation are further evidenced by increased displays of positive emotions in the presence of animals compared to toys. When allowed time with the guinea pigs, children smiled and laughed more often than they did with the toys. These findings are consistent with previous research, including a case study of a child with ASD who smiled more often during therapy sessions with a dog than without a dog [26] as well as a group-design study which demonstrated that children with ASD laughed more often in the presence of a therapy dog compared to a ball or stuffed dog [25]. It has been suggested that animals can lighten the mood and provide a humorous and positive focus for attention [60]. Indeed, the current results indicate that animals may provide a more powerful stimulus than toys for encouraging positive affect in social contexts for children with ASD.

The present study also provides the first evaluation of the valence of verbal content in the presence of animals versus toys. We found that children with ASD were more likely to make positive statements about liking things or being happy in the presence of animals compared to toys. They were also less likely to report sadness or discontent when with animals compared to toys. These outcomes may indicate a more positive mood when interacting with animals. Little research has been undertaken to examine the mood-enhancing effects of animals. The current findings suggest that further study regarding the ability of animals to increase positive emotional displays is warranted.

Another key finding from our study was that children with ASD displayed more prosocial behaviors towards humans in the presence of animals compared to toys. This outcome may be explained in part by the types of activities children engaged in during the sessions. For instance, during the animal sessions, children spent most of their time (78.5% of all 10-second intervals) doing things for the animal, such as feeding or grooming. By comparison, during the toy sessions, children spent most of their time (94.3% of all 10-second intervals) doing things for themselves, such as playing with a paddle-ball or blowing bubbles. The outward focus of caring for the animal may have carried over into awareness of the other humans who would benefit from help or assistance. Previous HAI studies have also suggested that TD children learn about prosocial behaviors through learning to care for and interact with animals [61]. Our results were also consistent with a recent study that showed increased prosocial behaviors following the introduction of a dog into the home [18].

Further investigation into the mechanisms for increased prosocial behaviors in the presence of animals may be useful to better understand the influence of animals on child socio-emotional development. Emerging research is beginning to reveal a relationship between the level of attachment to the animal and subsequent benefits received from interacting with the animal (e.g., [62]). In the present study, children with ASD demonstrated warmth and affection to the animals, but not to humans. This paradox may indicate that they felt more comfortable or closer to the animals than the people. Or, it may evidence a different type of relationship between children with ASD and animals versus children with ASD and other humans. In the current study, animal presence facilitated human-directed social approaches in addition to animal-directed affection. A better understanding of the relationship between attachment, affection, and socio-emotional outcomes from HAI may be helpful to foster improved social relationships for children with ASD in the future. In addition, it will be informative for further studies to directly assess differences in social behavior between ASD and TD children in the presence of animals versus toys to determine whether the effects of animal presence are greater for children with ASD or whether they are similarly effective for TD children.

The present study is limited by the lack of information regarding participant cognitive functioning or IQ. These factors might act as moderators of communicative outcomes and should be included in further studies of this nature. It is also difficult to determine which components of animal presence or animal interaction are responsible for the current results. Potential factors that may be implicated include the novel experience of animal interaction or the presentation of an engaging stimulus; however, these do not appear to be robust explanations for the results. For example, the effects of animal presence do not appear to be due to novelty effects of a new animal, given that the guinea pigs lived in the school classroom for eight weeks. There were also no differences in outcomes when comparing the first animal session to any toy sessions or the last animal session to any toy sessions. Further, the effects do not seem to be related to more engagement with animals than toys. Although most children preferred the guinea pigs to the toys, they did not appear to be vacant and bored during the toy sessions. Indeed, they made more physical contact with the toys than the animals and spent more time looking at the toys than the animals. However, they were not so enraptured by the toys that they neglected human contact. We found that children with ASD talked more to their peers (but not adults) and received more verbal social approaches (but not visual or physical) from their peers in the presence of toys compared to animals. These findings suggest that the toy condition provided an engaging and effective attention control for the animal condition. They also indicate that the social facilitation effect of the animals was not contingent solely on their presence as a novel or engaging stimulus. Instead, animals appear to contribute a unique component to social situations that encourages social interaction above and beyond the presence of something new, fun, and engaging.

In conclusion, findings from the current study provide evidence that children with ASD appear to demonstrate more social approach behaviors in the presence of animals compared to toys. These findings are of clinical value as they suggest that the inclusion of animals in therapeutic intervention, known as Animal-Assisted Intervention, may be an effective way to increase social interaction and enhance social behavioral outcomes. They also provide insight into a new strategy to increase interactions for children with ASD with their TD peers in the school classroom. Future studies should extend the current research on animal presence by evaluating the addition of targeted therapeutic protocols in order to maximize the socio-emotional and behavioral benefits of HAI.

## Supporting Information

Appendix S1 **Observation of Human-Animal Interaction for Research (OHAIRE).** Behavior coding definitions.
(DOCX)
