# Supplementary material for: Social Behaviors Increase in Children with Autism in the Presence of Animals Compared to Toys
Source: PLoS One. 2013 Feb 27;8(2):e57010. doi: 10.1371/journal.pone.0057010 (PMC3584132; doi:10.1371/journal.pone.0057010)
Supplement: Appendix S1 — Observation of Human-Animal Interaction for Research (OHAIRE). Behavior coding definitions. (DOCX) [file pone.0057010.s001.docx]

**Appendix S1**

*Observation of Human-Animal Interaction for Research (OHAIRE): Behavior Coding Definitions*

1 SOCIAL APPROACH BEHAVIORS

1.1 *Verbal*

1.1.a Speaking

The participant should be coded as speaking if ALL of the following conditions are met:

- - The participant’s lips are moving and/or there is audible sound when nobody else’s lips are moving.
  - The participant is speaking audible words, NOT making noises that are not words. For example, “uh oh” and “pop” are not words.
  - The participant is NOT engaging in verbal stereotypy (i.e., non-contextual words or phrases, or repetition of the same non-purposeful phrase). For example, do not code as talking if the participant is saying the same non-purposeful statement more than two times in a row without a response.

1.1.b Target

The target of the participant’s speech should recorded as (a) peer, (b), teacher, (c) animal, or (d) unknown, in accordance with the following definitions:

- - *Peer* or *Teacher*: The participant looks at the person during or immediately prior to speaking and/or the person responds to their speech. Responses can be verbal or facial (e.g., raising eyebrows or watching participant speak).
  - *Animal*: The participant is looking at the animal while speaking and addresses the animal directly (e.g., saying “you” or the animal’s name).
  - *Unknown*: The target is not the animal and no humans respond.

1.1.c Topic

The topic of the participant’s speech should be recorded as (a) toy, (b) animal, (c) other, or (d) unknown based on the content of their speech and/or resultant responses. If the spoken words are difficult to decipher or incoherent, the topic should be recorded as “unknown.”

1.1.d Valence

The valence of the participant’s speech should be recorded as (a) positive, (b) negative, or (c) neutral, in according to the following definitions:

- - *Positive*: Speech has an overtly positive tone and/or a concurrent smile (e.g., expressions of joy, liking, or happiness).
  - *Negative*: Speech has an overtly negative tone and/or concurrent frown (e.g., whining, complaining, disliking, sadness).
  - *Neutral*: Speech is neither positive nor negative (e.g., a non-rhetorical question).

1.2 *Visual*

1.2.a Looking

The participant’s gaze should be assessed by the angle and direction of their face. Targets of looking include (a) peer, (b) teacher, (c) toy, and (d) animal.

- - *Peer* or *Teacher:* Human-directed gaze should be coded as looking only if the gaze is in the direction of a human’s face.
  - *Toy* or *Animal:* Toy- and animal-directed gaze should be coded as looking if the gaze is in the direction of any part of the object or animal.
  - If the participant is not looking at anything or anyone, do not code “looking.”

1.3 *Physical*

1.3.a Touching

Touching should be coded if the participant makes physical contact with the other participants or with an experimental object. Do not code as touching if there is concurrent aggression or problem behavior involved. Targets of touching include (a) peer, (b) teacher, (c) toy, or (d) animal.

- - *Peer* or *Teacher:* Touching is coded when any part of the participant’s body makes contact with any part of the target’s body. This code will predominantly demonstrate physical proximity (e.g., two participants sitting next to each other with knees touching).
  - *Toy* or *Animal:* Touching is coded when one or both of the participant’s hands touch the object. These codes demonstrate physical engagement with the toy or animal.

1.3.b Affection

Affection should be coded if the participant demonstrates physical affection to other participants or an experimental object. Examples of affection include, but are not limited to: hugging, cuddling, nuzzling, comforting, and/or petting. Targets of affection include (a) peer, (b) teacher, (c) toy, or (d) animal.

- - *Peer* or *Teacher:* Affection is human-directed (e.g., hugging or comforting a peer).
  - *Toy* or *Animal:* Affection is toy-directed (e.g., cuddling or hugging a doll) or animal-directed (e.g., cuddling or petting the animal).

2 BEHAVIORAL INTENTIONS

2.1 *Self-focused behavior*

2.1.a Self-focused activity

Self-focused activity should be coded if the participant is isolated in that they are engaging in play of self-stimulatory behaviors directed to the self. For example, the participant is engaging in solitary toy play or drawing a picture of the guinea pigs.

2.2 *Prosocial behavior*

2.2.a Other-focused activity

Other-focused activity should be coded if the participant engages in prosocial behavior by doing something for another person or for the animal. This code is the contrast to self-focused activity (2.1.b) in which the target of the activity is the participant themselves, rather than another person or animal. Targets of other-focused activities include (a) person or (b) animal.

- - *Person:* Examples of other-focused activities for another person include providing assistance, helping, offering to do a favor, or handing a material.
  - *Animal:* Examples of other-focused activities for the animal include care-taking activities for the animal such as feeding, brushing, holding, or cleaning.

3 PROBLEM BEHAVIORS

3.1 Aggression

Aggression should be coded if the participant engages in verbal or physical behavior that injures or irritates another participant. Verbal aggression includes mean or derogatory comments. Targets of aggression include (a) peer, (b) teacher, (c) toy, or (d) animal.

3.2 Disruption

Disruption should be coded if the participant is engaging in silly or playful behavior that is disruptive to the other participants. For example, this code should be used if a participant is making loud jokes that disturb other people in the group who are trying to have a conversation.

3.3 Stealing

Stealing should be coded if a participant takes (or attempts to take) a toy or animal from another person. This code should also be used if the participant is pestering the other child to give them the desired toy or animal (i.e., asking for something more than twice within the same 10-second interval).

3.4 Leaving

Leaving should be coded when the participant is not sitting or standing within the defined session area. The area in the current study is defined as behind the session materials (i.e., toys or animal enclosure) or just outside the materials if additional space is required for the desired activity (e.g., moving aside to use ping pong paddle so as not to hit other participants).

3.5 Other problem behaviors

Other problem behaviors should be used to designate any other problematic behaviors which do not fit into the above categories. It includes any action that is not calm or productive, such as running around, crying, screaming, banging, destruction, ignoring facilitator instructions, playful hitting (either with hand or object) that is not full aggression.

4 EMOTIONAL DISPLAY

4.1 *Positive emotions*

4.1.a Smiling

Smiling should be coded when the participant demonstrates a change in facial expression by turning up the corners of the mouth and/or spreading the lips.

4.1.b Laughing

Laughing should be coded when the participant engages in audible laughter. The laughing may be soft or hard and with or without vocalization. The minimum requirement for vocalized laughter is one laugh, whereas the minimum requirement for non-vocalized laughter (i.e., breath laughter) is two laughs (in order to distinguish laughter from heavy breathing or sniffling).

4.2 *Negative emotions*

4.2.a Frowning

Frowning should be coded when the participant demonstrates a change in facial expression by turning down the corners of the mouth and/or tightening the lips.

4.2.b Crying

Crying should be coded if the participant engages in an audible cry. Crying can occur with or without tears, but requires scrunching up of the face in addition to a vocalization.

4.2.c Whining

Whining should be coded if the participant makes whining or complaining vocalization without scrunching up the face as in crying.

4.2.d Pain

Pain should be coded if the participant makes a noise (e.g., “ouch”) to indicate physical pain. *Note:* In the present study, pain was included in the frown/cry/whine composite score.

**Access to the internet-based OHAIRE data collection instrument can be obtained by contacting the authors.
